# Could Nutraceutical Approaches Possibly Attenuate the Cytokine Storm in COVID-19 Patients?

**DOI:** 10.3389/fcimb.2021.667733

**Published:** 2021-04-23

**Authors:** Ramachandran Vignesh, Vijayakumar Velu, Sripathi M. Sureban

**Affiliations:** ^1^ Preclinical Department, Royal College of Medicine Perak (UniKL RCMP), Universiti Kuala Lumpur, Ipoh, Malaysia; ^2^ Infectious Diseases Laboratory, YR Gaitonde Centre for AIDS Research and Education (YRG CARE), Chennai, India; ^3^ Department of Pathology & Laboratory Medicine, Emory University School of Medicine, Division of Microbiology & Immunology, Yerkes National Primate Center, Atlanta, GA, United States; ^4^ Digestive Diseases and Nutrition Section, Department of Internal Medicine, The University of Oklahoma Health Sciences Center, Oklahoma City, OK, United States

**Keywords:** COVID-19, gut microbiota, nutraceuticals, probiotics, Pectin, SARS-CoV-2

## Introduction

Coronavirus disease 2019 (COVID-19) caused by the severe acute respiratory syndrome coronavirus 2 (SARS-CoV-2) has had a colossal impact on public health, testified by being announced as a global pandemic by the World Health Organization (WHO) in March 2020. As of 1^st^ April 2021, there have been over 128,223,872

confirmed cases of COVID-19 and over 2,804,120 fatalities reported by WHO (WHO Coronavirus Disease (COVID-19) Dashboard, 2021). Though COVID-19 vaccines are developed and being deployed around the world, considering the logistic challenges, vaccine-induced herd immunity is still a long way off (Vignesh et al., 2020). Despite being primarily a respiratory disease, mounting pieces of evidence point towards the impact of COVID-19 on the gastrointestinal system and the presentation of gastrointestinal (GI) manifestations (McDermott et al., 2020). In this opinion article, we summarize the effects of COVID-19 on the GI tract and also provide evidence of the role of nutraceuticals as a potential treatment strategy against COVID-19.

## Coronavirus Disease 2019 (COVID-19) in Gastrointestinal Tract (GIT)

Coronaviruses, the single-stranded RNA viruses are spherical in shape and of their key structural components, are comprised of key structural components namely, the spike (S), envelope, membrane, and nucleocapsid proteins. Of these, the S protein remains the main determinant of pathogenicity being a requisite for viral entry into host cells. For the SARS-CoV-2 to gain entry into the host cell, it utilizes the receptor angiotensin-converting enzyme 2 (ACE2) and for S protein priming it uses the transmembrane serine protease 2 (TMPRSS2). Following entry, the replication of SARS-CoV-2 occurs in various cell types based on the expression of the ACE2 receptors such as in the lung cells, epithelial cells, enterocytes, and hepatocytes (Ferreira et al., 2020). Studies have revealed that the expression of ACE2 in the small intestine and colon is about 40x and 3x respectively higher relative to the lungs. Likewise, concerning the lungs, expression of TMPRSS2 is about 2x and 20x more in the small intestine and colon respectively (Cardinale et al., 2020; Zhang et al., 2020). Thus, the virus eventually produces and assembles new virions inside the cell which get released into the GIT. Studies have established the shedding of SARS-CoV-2 in the GIT even after the resolution of respiratory symptoms, underscoring the implications of GI infection. Albeit not as common as respiratory symptoms, GI symptoms like anorexia, nausea, vomiting, and diarrhea have been observed in patients with COVID-19. Meta-analysis studies have reported a pooled prevalence of GI symptoms among COVID-19 patients at about 18% (Cheung et al., 2020; Zhao et al., 2020). GI symptoms are observed to be associated with inflammatory processes leading to intestinal damage. Studies have revealed that by causing ACE2 modifications, SARS-CoV-2 infection in the gut could lead to gastrointestinal inflammation and other manifestations like diarrhea (Hoffmann et al., 2020). A study has demonstrated the role of SARS-CoV-2 infection in inflammatory reactions in the GIT as evidenced by diarrheal symptoms, increased levels of fecal calprotectin (FC), and a systemic IL-6 response (Effenberger et al., 2020).

The paramount role of the mucosal immune system has been implicated in the pathogenesis of COVID-19 at several levels (Russel et al., 2020). The secretory IgA antibodies have a key role in the protection of mucosal surfaces in the lung and gut from pathogenic viruses *via* various mechanisms. Recent findings suggest the predominance of secretory IgA antibodies in SARS-CoV-2 specific early humoral responses and these IgA antibodies have also exhibited relatively better-neutralizing activity than that of IgG (Sterlin et al., 2021). Interestingly, very high titers of SARS-CoV-2-specific IgA have been observed to correlate with the severe acute respiratory syndrome (Cervia et al., 2021). Also, significantly higher titers of IgA with potent neutralizing ability have been observed among COVID-19 patients with gastrointestinal symptoms indicating the key role of IgA (Wang et al., 2021). With mounting evidence from studies demonstrating the cross-talk between the lungs and gut microbiota, secretory IgA could be a significant mediator of this ‘gut-lung axis’ (Sencio et al., 2021; Vignesh et al., 2021).

## Pathogenesis Mechanisms of Cytokine Storm in COVID-19

### Dysregulation of Renin-Angiotensin System (RAS)

Various studies have demonstrated that the modulation of systemic inflammation is caused by regulating the renin-angiotensin system (RAS) that includes the ACE2. Homeostasis of RAS-ACE2 is required for healthy conditions and an imbalance to this is observed in the diseased conditions including diabetes, hypertension, and cardiovascular disorders (Villapol and Saavedra, 2015; Hoffmann et al., 2020). Interestingly, downregulation of ACE2 levels in tissues is associated with the pathogenicity of the virus leading to an imbalance of positive and negative regulation of RAS (Dijkman et al., 2012). It is already known that ACE-2 receptor blockers have been known to be effective in the management of diabetes, cardiovascular diseases, renal, and metabolic disorders (Villapol and Saavedra, 2015; Garg et al., 2020). Reduced expression of ACE2 has been observed to be associated with various conditions such as hypertension, diabetes, and cardiovascular conditions, which are also associated with COVID-19 as comorbidities (Magrone et al., 2020). Studies have shown upregulation of RAS by SARS-CoV-2 thereby depleting ACE2 in cardiovascular patients (Villapol, 2020). Thus, in COVID-19, perturbation of RAS-ACE2 balance could worsen the inflammatory responses leading to severe COVID-19 outcomes in patients with pre-existing comorbidities. In addition to its role in intestinal inflammation, ACE2 also has a key effect on the composition of the intestinal microbiota (Cole-Jeffrey et al., 2015), thereby hinting at the possible link for perturbed gut microbiota in the severity of COVID-19 among patients with pre-existing comorbidities.

## The Possible Role of Gut Dysbiosis and Associated Leaky Gut

Metabolites of gut microbial flora such as the short-chain fatty acids (SCFAs) play a significant role in the modulation of immune and inflammatory responses (Gonçalves et al., 2018) These SCFAs can efficiently minimize the exaggerated inflammatory responses by augmenting the functions of T helper cells, regulatory cells, and Th17 effector cells (Li et al., 2018). They are also pivotal in maintaining the integrity of gut epithelium, thereby preventing leakage and eventual microbial translocation.

Gut dysbiosis refers to an alteration in the composition of gut microbiota wherein the normal flora gets replaced by pathogenic microbes. This may be caused by a plethora of factors including aging and is associated with the pathogenesis of various diseases (Donati Zeppa et al., 2020). Various studies demonstrating the role of gut dysbiosis in aging-related cardiovascular, metabolic, and renal disorders, correlates with the increased severity of COVID-19 observed in patients with these pre-existing conditions (Chiappetta et al., 2020; Sanchez-Rodriguez et al., 2020; Vignesh et al., 2021). A recent fecal metabolomic study demonstrated the possible link between the gut microbiota and inflammatory responses leading to severe COVID-19 manifestations.(*Gou: Gut microbiota may underlie the predisposition… - Google Scholar*, no date) Several studies have also reported the reduction in the relative abundance of beneficial bacteria in patients with COVID-19, thereby supporting the concept of gut dysbiosis (Gou et al., 2020; Gu et al., 2020; Zuo et al., 2020).

Dysbiosis of the gut has been demonstrated to correlate with lowered production of gut bacteria-derived metabolites such as butyrate, leading to increased gut permeability (Mosca et al., 2016). When the integrity of the gut barrier is compromised, it facilitates the translocation of microbial-derived products thereby activating the immune system and triggering inflammatory responses (Gunness and Gidley, 2010; Fernandes et al., 2019). This leaky gut phenomenon could also possibly explain the disseminated spread of SARS-CoV-2 from the gut to other organs expressing ACE-2 (Kim, 2021).

## Foraying Through the Eye of Cytokine Storm

Extreme systemic hyperinflammatory symptoms triggered by ebullient immune responses are characteristic of the cytokine storm observed in severe COVID-19 patients. Various observations point towards an interplay of multiple factors in the pathogenesis of COVID-19 induced cytokine storm syndrome. Several studies have drawn parallels between the pathophysiology of cytokine storm syndrome in severe COVID-19 and the acute radiation syndrome (ARS) with inflammation being the common key player (Rios et al., 2021). Cytokine storm has been characterized by the elevated serum levels of the following cytokines and chemokines such as interleukin-1β (IL-1 β), IL-2, IL-7, IL-8, IL-9, IL-10, granulocyte colony-stimulating factor (G-CSF), granulocyte-macrophage colony-stimulating factor (GM-CSF), Interferon-gamma (IFN-*γ*), tumor necrosis factor-alpha (TNF-α), IFN-*γ*- inducible protein-10 (IP-10), monocyte chemoattractant protein-1 (MCP1) and macrophage inflammatory protein-1 A (MIP1A) (Wu and Yang, 2020). Studies indicate an active role of Th17 lymphocytes as evidenced by elevated secretion of various pro-inflammatory cytokines and interestingly it is the same systemic inflammatory reaction observed in the case of intestinal microbial translocation (Cardinale et al., 2020; Wu and Yang, 2020).

In various viral infections, the role of receptors of pathogen-associated pattern molecules like the lipopolysaccharides (LPS) and toll-like receptor 4 (TLR-4) have been implicated in the induction of inflammatory reactions. It is cardinal to note the possibility of the key role of LPS, during microbial translocation due to leaky gut (Olejnik et al., 2018).

Besides, in the pathogenesis of severe COVID-19, there is also an implication of the cross-talk between the gut barrier and lung through the gut-lung axis (Openshaw, 2009; Vignesh et al., 2021).

Thus, the activation of innate and adaptive immune responses along with the role of complement could orchestrate the cytokine storm eventually leading to acute respiratory distress syndrome (ARDS) and multiple organ failure.

## Nutraceutical Approaches for Attenuation of Inflammatory Responses

### Probiotics

Probiotics comprising mainly of the lactic acid bacterial strains are known to modulate the gut microbiota by suppressing other pathogenic bacteria. The probiotics have been shown to possess anti-inflammatory and immune-modulatory properties (Akour, 2020; Khaneghah et al., 2020). Mounting shreds of evidence from several *in vitro* studies suggest that the active anti-inflammatory role of probiotics by suppression of pro-inflammatory cytokine expression and reduction in the release of inflammatory mediators (Schmitter et al., 2018; Yu et al., 2019).

Animal experiments revealed upregulation of anti-inflammatory cytokine genes and levels of IL-10 and upregulation of anti-inflammatory cytokine genes and levels of IL-6 in probiotic-treated rats. Clinical studies have demonstrated a reduction of c-reactive protein and IL-6 levels in sera and pro-inflammatory biomarkers in the gastrointestinal tract upon probiotic administration (Plaza-Díaz et al., 2017; Morshedi et al., 2019). Studies have been reported that the role of probiotics in minimizing the occurrence and duration of viral respiratory infections (Hao et al., 2011; King et al., 2014). Several studies have revealed the dysbiosis of gut microbiota in COVID-19 patients with a relatively decreased proportion of beneficial bacteria. Studies have documented the role of probiotics in making COVID-19 patients less susceptible to secondary infections (Mak et al., 2020).

**Figure 1:**
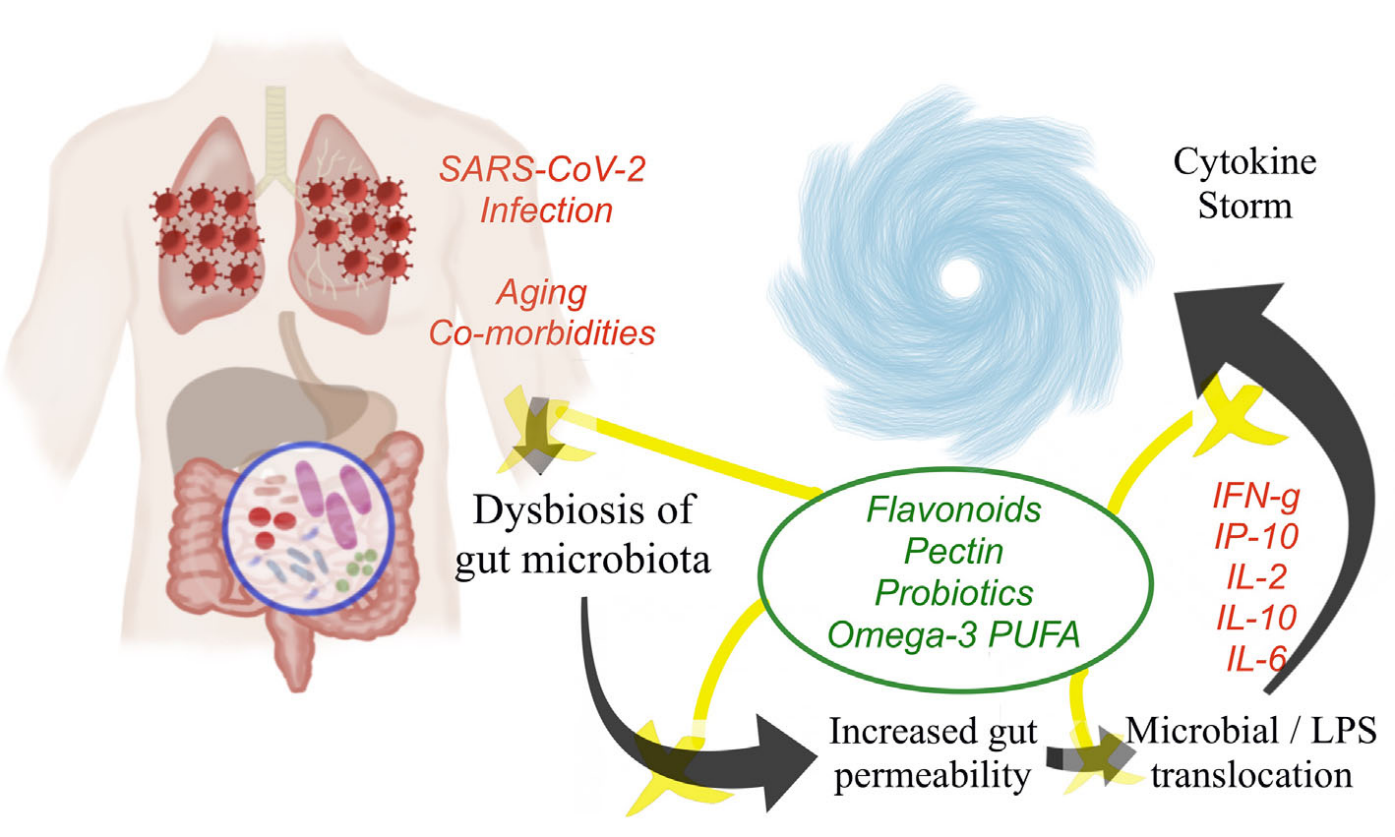
Pathophysiological events of COVID-19 leading to cytokine storm and the potential role of nutraceuticals in attenuating the cytokine storm.

Though there is no concrete evidence on the possible nutraceutical intervention by probiotics in COVID-19 patients, probiotics could prevent or restore the SARS-CoV-2-induced gut mucosal damage and inflammation through one of the various beneficial mechanisms discussed. **Figure 1** represents the schematic representation of the pathophysiological events of COVID-19-induced cytokine storm and the potential role of nutraceuticals in minimizing the exaggerated inflammatory responses.

### Flavonoids

Flavonoids are products of plant metabolism and have been known to possess potent antioxidant properties. Also, they have been demonstrated to have other biological functions like anti-inflammatory and immune modulation thereby generating interest (Pérez-Cano and Castell, 2016). Several flavonoids have been investigated for potential antiviral properties based on several *in vitro* and *in vivo* studies. Flavonoids such as luteolin and kaempferol derivatives have demonstrated antiviral properties against SARS-CoV-2 and other viruses by interfering with the viral replication (Yu et al., 2012; Zakaryan et al., 2017). The flavonoids also possess the ability to modulate the gut microbiota and are known to promote the growth of beneficial bacteria while minimizing the growth of pathogenic microorganisms (Carrera-Quintanar et al., 2018; Kumar Singh et al., 2019) Flavonoids also play a key role in the regulation of intestinal immune homeostasis and reduction of exotoxins thereby improving gut health (Pei et al., 2020).

Pectin fiber-rich in galactoside and hesperidin-rich citrus pectin have been studied to have several beneficial activities such as free radical scavenging and reduction of systemic inflammation (Voragen et al., 2009; Ciriminna et al., 2020; Meneguzzo et al., 2020). Animal experiments have revealed the role of dietary pectin in the promotion of overall survival among mice exposed to ionizing radiation (IR) compared to the control group (Sureban et al., 2015). Antiviral activity of hesperidin has been demonstrated against viruses such as influenza and herpes. Recent computational studies have demonstrated a significant binding affinity of hesperidin to the three key protein receptors of SARS-CoV-2 namely, the SARS-CoV-2 protease domain, the receptor-binding domain of spike glycoprotein (RBD-S), and the receptor-binding domain of ACE2. Molecular docking studies have reiterated this high binding ability of hesperidin and in this aspect, it was observed to even outperform several natural products and even the antiviral drug lopinavir (Meneguzzo et al., 2020; Utomo et al., 2020). These findings suggest the possible nutraceutical role of pectin rich in flavonoids and hesperidin to attenuate the cytokine storm in severe COVID-19 cases.

## Omega-3 Polyunsaturated Fatty Acids

Omega-3 polyunsaturated fatty acids (Omega-3 PUFAs), abundantly present in fish and flaxseed oils are known to have effects on the immune system by influencing macrophages and modulating immune responses as demonstrated by various *in vitro* and *in vivo* studies (Calder, 2013; Parolini, 2019). Omega-3 PUFAs have been demonstrated to have a pivotal role in modulating cytokine and chemokine production by the macrophages and also be metabolized by various immune regulatory metabolites such as prostaglandins and leukotrienes (Gutiérrez et al., 2019). Studies have demonstrated enhanced intestinal crypt survival in radiation-exposed mice treated with dietary fish oil rich in Omega-3 PUFAs (Sureban et al., 2016). Animal experiments have pieces of evidence suggesting their critical role in maintaining gut mucosal integrity by their interaction with the gut microbiota (Costantini et al., 2017). Interestingly, studies have also shown an increase in SCFA-producing beneficial gut microflora upon dietary intake of Omega-3 PUFA (Patterson et al., 2014; Watson et al., 2018).

Omega-3 fatty acids such as eicosapentaenoic acid (EPA) and docosahexaenoic acid (DHA) have been extensively studied to possess various beneficial effects such as being anti-inflammatory and reducing coagulation. EPA and DHA are known to replace arachidonic acid in the phospholipid membranes, which is known to increase reactive oxygen species. In addition, by contributing to the synthesis of less inflammatory eicosanoids and specialized pro-resolving lipid mediators like resolvins and protectins, EPA and DHA efficiently reduce intestinal and systemic inflammation (Rogero et al., 2020). A pilot study involving 100 COVID-19 patients set out to assess the relationship between the blood EPA+DHA levels and risk of mortality reported a strong trend and likelihood of EPA and DHA lowering the risk of death up to 75% due to COVID-19 (Asher et al., 2021).

## Discussion

With the COVID-19 pandemic still being rampant and continuing to be a global challenge to mankind, being vigilant and strategizing for alternative complementary approaches for better outcomes in susceptible populations is of paramount importance. Novel and safe nutraceutical approaches to attenuate the exaggerated inflammatory responses by modulation of gut microbiota seem to be the way forward to optimize COVID-19 outcomes.

## Author Contributions

RV, SS and VV led the writing of this opinion article. All authors contributed to the article and approved the submitted version.

## Funding

VV was supported by National Institutes of Health Grants 1R01AI148377-01A1, R01 HD095741-01, CFAR R03 (to VV), Emory University CFAR grant P30 AI050409 and NCRR/NIH base grants P30 RR00165, P51OD011132 (to Y.N.P.R.C).

## Conflict of Interest

SS is an inventor on several patents with a commercial interest and also has an ownership interest in COARE Holdings Inc.

The remaining authors declare that the research was conducted in the absence of any commercial or financial relationships that could be construed as a potential conflict of interest.
